# RUNX3 Regulates Intercellular Adhesion Molecule 3 (ICAM-3) Expression during Macrophage Differentiation and Monocyte Extravasation

**DOI:** 10.1371/journal.pone.0033313

**Published:** 2012-03-29

**Authors:** Ana Estecha, Noemí Aguilera-Montilla, Paloma Sánchez-Mateos, Amaya Puig-Kröger

**Affiliations:** 1 Laboratorio de Inmuno-Oncología, Instituto de Investigación Sanitaria Gregorio Marañón, Hospital General Universitario Gregorio Marañón, Madrid, Spain; 2 Centro de Investigaciones Biológicas, CSIC, Madrid, Spain; University of Regensburg, Germany

## Abstract

The adhesion molecule ICAM-3 belongs to the immunoglobulin gene superfamily and functions as a ligand for the β2 integrins LFA-1, Mac-1 and α_d_β_2_. The expression of ICAM-3 is restricted to cells of the hematopoietic lineage. We present evidences that the ICAM-3 gene promoter exhibits a leukocyte-specific activity, as its activity is significantly higher in ICAM-3+ hematopoietic cell lines. The activity of the ICAM-3 gene promoter is dependent on the occupancy of RUNX cognate sequences both *in vitro* and *in vivo*, and whose integrity is required for RUNX responsiveness and for the cooperative actions of RUNX with transcription factors of the Ets and C/EBP families. Protein analysis revealed that ICAM-3 levels diminish upon monocyte-derived macrophage differentiation, monocyte transendothelial migration and dendritic cell maturation, changes that correlate with an increase in RUNX3. Importantly, disruption of RUNX-binding sites led to enhanced promoter activity, and small interfering RNA-mediated reduction of RUNX3 expression resulted in increased ICAM-3 mRNA levels. Altogether these results indicate that the ICAM-3 gene promoter is negatively regulated by RUNX transcription factors, which contribute to the leukocyte-restricted and the regulated expression of ICAM-3 during monocyte-to-macrophage differentiation and monocyte extravasation.

## Introduction

Intercellular Adhesion Molecule 3 (ICAM-3, CD50) is a cell surface molecule which belongs to the immunoglobulin gene superfamily, and whose extracellular region contains five immunoglobulin-like domains. Originally identified as a molecule involved in lymphoblastoid cell adhesion to purified LFA-1 (CD11a/CD18) [1], numerous studies have now provided evidence that ICAM-3 functions as a ligand for LFA-1, Mac-1 (CD11b/CD8) and α_d_β_2_ integrins [2], [3], [4]. Moreover, ICAM-3 has been also proposed as a ligand for the Dendritic Cell-Specific ICAM-3-Grabbing Non Integrin (DC-SIGN) C-type lectin [5]. Whereas LFA-1 interacts with the most N-terminal immunoglobulin domain of ICAM-3 (domain I) [6], the second domain of recombinant ICAM-3 is responsible for the interaction with high mannose-containing carbohydrates of DC-SIGN [7].

From the functional point of view, ICAM-3 mediates a plethora of immunologically relevant homotypic and heterotypic intercellular interactions [2], [8], such as leukocyte recruitment during migration [9], removal of apoptotic cells [10] and lymphocyte interactions with antigen-presenting cells [11]. Importantly, ICAM-3 is involved in the interactions that take place during the early stages of the immunological synapse stablishment [12]. ICAM-3 engagement on the T cell surface increases the CD3-mediated up-regulation of CD25 and CD69 [13] and initiates intracellular signaling including calcium transients [14], [15] and tyrosine phosphorylation [16]. In addition to its role in leukocyte adhesion, ICAM-3 also contributes to leukocyte migration by virtue of its relocalization to the trailing edge upon leukocyte polarization [9], [17], an effect that takes place by its interaction with cytoskeletal components such as ERM proteins [17]. Consequently, ICAM-3 is not only a cell surface adhesion molecule but functions as a co-stimulatory molecule with intracellular signaling capability.

In spite of the critical effector functions mediated by ICAM-3, the molecular mechanisms underlying its expression have not yet been characterized. ICAM-3 is structurally and functionally homologous to the LFA-1 ligands ICAM-1 and ICAM-2, but exhibits a different pattern of expression. While ICAM-1 expression is ubiquitous and activation-dependent [18] and ICAM-2 is endothelial cell-specific [19], the expression of ICAM-3 is restricted to cells of the hematopoietic lineage [6]. More specifically, ICAM-3 is found on the surface of most leukocytes and is highly expressed on peripheral blood granulocytes, monocytes and lymphocytes [6]. Unlike ICAM-1, the cell surface expression of ICAM-3 is not dependent on the state of cellular activation, although higher ICAM-3 levels are seen in memory than on naïve T lymphocytes [15]. We now report that ICAM-3 protein and mRNA levels are dramatically reduced upon monocyte to macrophage differentiation and following monocyte transendothelial migration. To identify the *cis*-acting elements and transcription factors that control this regulation, we have functionally dissected the proximal regulatory region of the ICAM-3 gene. Our results indicate that the ICAM-3 gene promoter exhibits a leukocyte-specific activity that is dependent on the occupancy of RUNX and C/EBP cognate sequences both *in vitro* and *in vivo*. Moreover, RUNX factors transactivate the ICAM-3 promoter either alone or in collaboration with Ets-1 and C/EBPα factors. Finally, we also demonstrate that RUNX3 exerts a negative regulatory effect on ICAM-3 expression in myeloid cells. These results constitute the first description of transcription factors actively implicated in determining the leukocyte-specific and differentiation-regulated expression of ICAM-3.

## Results

### Tissue specific activity of the ICAM-3 gene promoter

**Figure 1:**
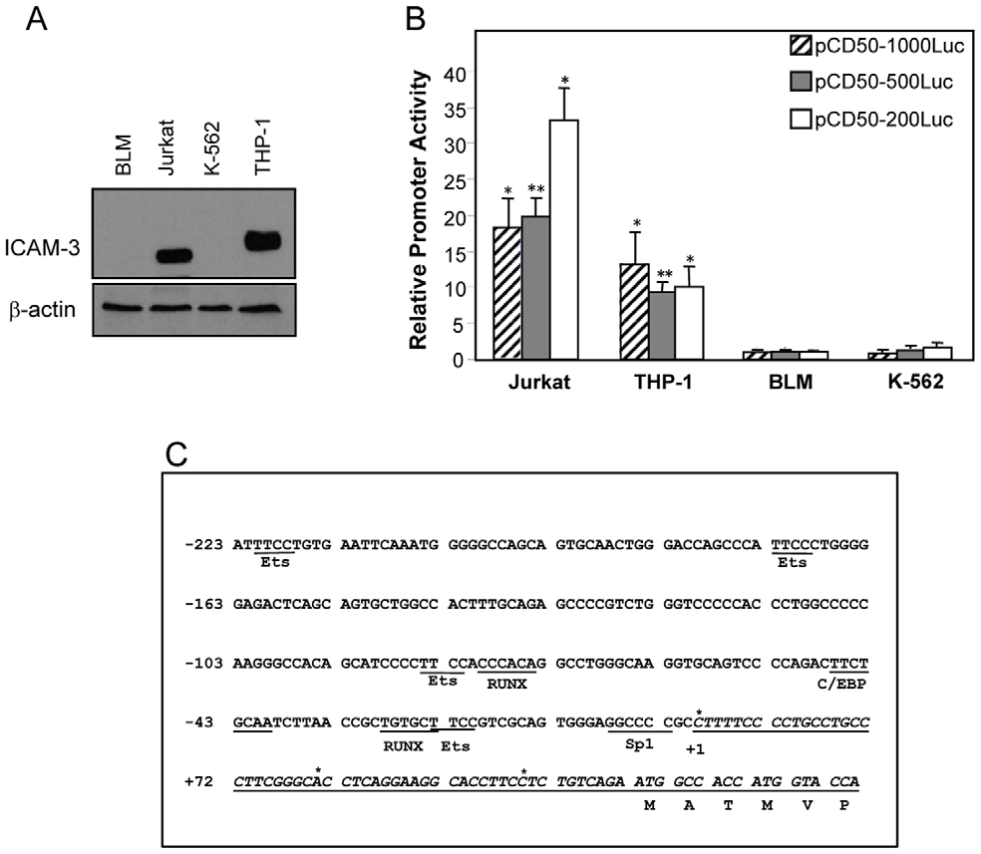
Restricted expression of ICAM-3 and cell-specific activity of the ICAM-3 promoter. **A.** Determination of ICAM-3 expression in BLM, Jurkat, K-562 and THP-1 cell lines by Western blot. As a control, β-actin expression levels were also determined. The experiment was performed twice and one of the experiments is shown. **B.** The ICAM-3 promoter-based constructs pCD50-1000, pCD50-500 and pCD50-200 were transfected in Jurkat (ICAM-3+), THP-1 (ICAM-3+), BLM (ICAM-3−) and K-562 (ICAM-3−) cell lines. After 48 hours cells were lysed and luciferase activity determined. For each reporter construct, promoter activity is expressed relative to the activity produced by the reporter plasmid in BLM cells, arbitrarily set to 1, after normalization for transfection efficiency. Data represent mean ± SD of 4 independent experiments using two different DNA preparations. (*p<0,05 for pCD50-1000Luc in THP-1 and Jurkat and p = 0.8 for K-562 when compared with the activity of pCD50-1000Luc in BLM cells; **p<0.005 for pCD50-500Luc in THP-1 and Jurkat and p = 0.5 for K-562 when compared with the activity of pCD50-500Luc in BLM cells; and * p<0.05 for pCD50-200Luc in THP-1 and Jurkat cells and p = 0.2 for K-562 cells when compared with the activity of pCD50-200Luc in BLM cells). **C.** Nucleotide sequence of the 5′-regulatory region of the ICAM-3 gene. The transcriptional initiation sites are identified by * and the major transcription initiation site is denoted by +1. First exon nucleotides are shown in boldface type and are underlined. The derived amino acid sequence is shown under the coding region of the first exon. Underlined areas correspond to consensus sequences for RUNX, C/EBP and Ets transcription factors.

The adhesion molecule ICAM-3 is the major ICAM expressed by resting leukocytes [6]. ICAM-3 is expressed by THP-1 myeloid cells and Jurkat lymphoid cells but not by erythroleukemic or melanoma cell lines like K-562 or BLM cells (Fig. 1A). The distinct mobility of the ICAM-3 on THP-1 and Jurkat cells probably reflects the cell type-specific glycosylation of the molecule, that appear to have functional consequences [20]. To determine the factors controlling ICAM-3 expression, we first sought to analyze the DNA elements and transcription factors involved in the leukocyte restricted-expression of ICAM-3. To that end, we amplified by PCR a genomic region immediately upstream of the structural region of the ICAM-3 gene (−1080/+53, pCD50-1000Luc) and generated two additional deletion constructs spanning the regions −505/+53 and −164/+53 (pCD50-500Luc and pCD50-200Luc). The three ICAM-3 promoter-based constructs were analyzed in ICAM-3+ THP-1 and Jurkat cells and the ICAM-3− K-562 and BLM cell lines (Fig. 1A). The three constructs exhibited higher activity in Jurkat and THP-1 ICAM-3+ cells than in K-562 or BLM cells (Fig. 1B). On average, the activity of the pCD50-1000Luc, pCD50-500Luc and pCD50-200Luc constructs was 25- fold higher in ICAM-3+ Jurkat cells than in non-expressing cells, and 14-, 9- and 10- fold higher in ICAM-3+ THP-1 cells than in non-expressing BLM cells. Therefore, the ICAM-3 proximal promoter displays tissue-restricted activity and exhibits a higher activity in cells with a constitutive expression of ICAM-3.

The sequence analysis of the region −164/+53 revealed that the ICAM-3 gene promoter lacks TATA and CCAAT boxes. 5′RACE allowed us to identify three major transcriptional start sites within the ICAM-3 gene in lymphoid cells, two of them conserved in myeloid cells. The major transcription initiation site (74% of the transcripts in Jurkat and 90% of the transcripts in THP-1 cells) was found 54 bp upstream from the translational start site, and coincides with the initial nucleotide of the predicted exon 1 (adscribed the +1 position, Fig. 1C). The sequence around the +1 position showed homology to the Initiator promoter element as it conforms to the consensus YYANWYY [21]. In Jurkat cells, two other transcriptional start sites were found 10 bp and 29 bp upstream from the first ATG and each one of them was used in 13% of the mRNA transcripts while in THP-1 cells 10% of the transcripts begin 10 pb upstream from the first ATG (Fig. 1C).

### RUNX1 and RUNX3 recognizes the ICAM-3 promoter *in vitro* and *in vivo*

**Figure 2:**
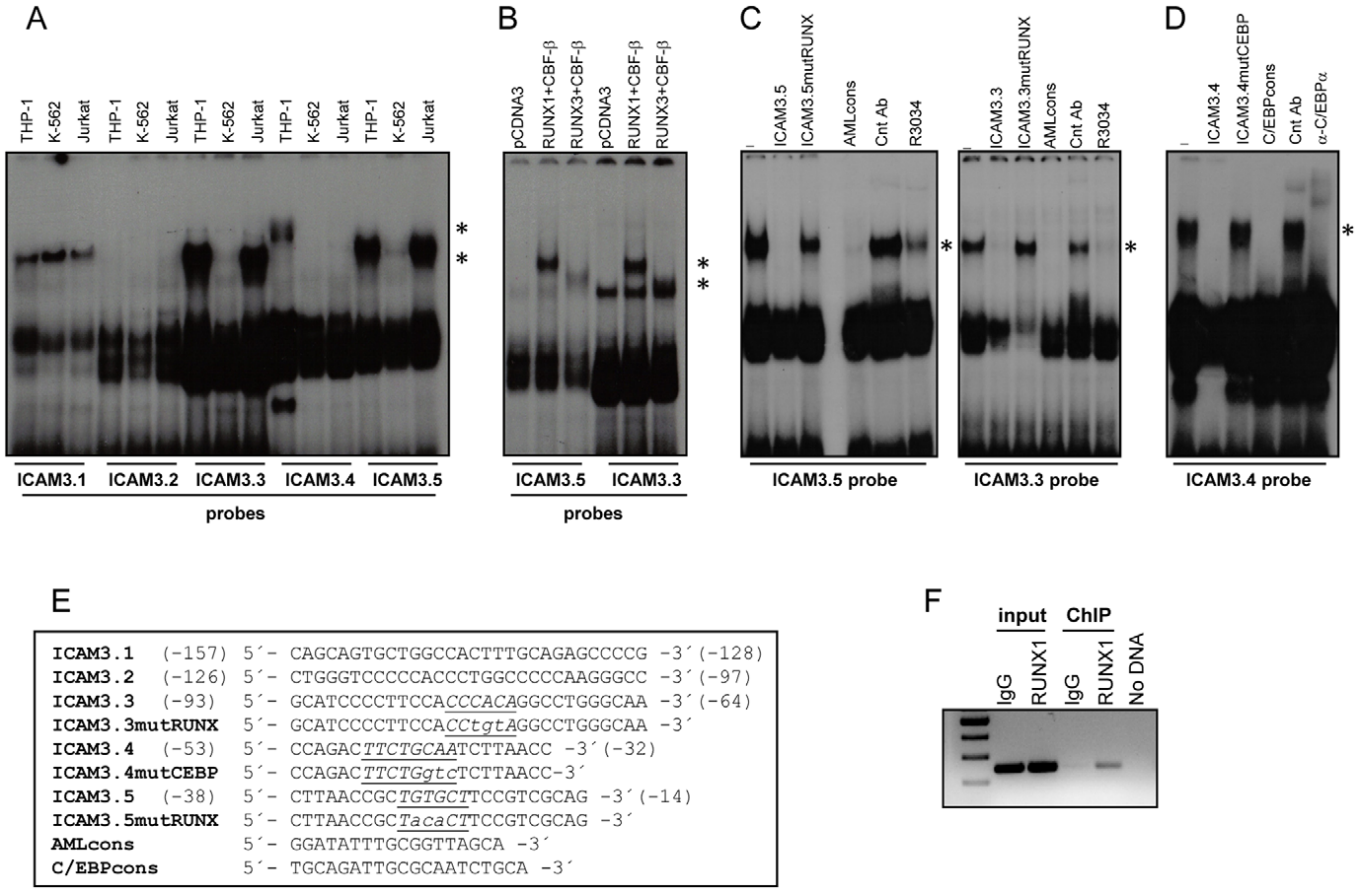
Identification and characterization of RUNX and C/EBP-binding elements within the ICAM-3 gene proximal regulatory region. **A.** EMSA was performed on the indicated oligonucleotides spanning the −157/−14 region of the ICAM-3 promoter using nuclear extracts from THP-1, K-562 and Jurkat cells. The position of the major retarded species is indicated. **B.** EMSA was performed on the ICAM3.3 and ICAM3.5 oligonucleotides using nuclear extracts from the indicated COS-7 cells transfected with an empty expression vector (pCDNA3) or with either RUNX1 or RUNX3 together with CBF-β expression vector. The position of the RUNX1- and RUNX3-containing complex is shown. **C.** EMSA was performed on the ICAM3.5 and ICAM3.3 oligonucleotides using nuclear extracts from Jurkat cells in the absence (−) or presence of unlabeled competitor oligonucleotides (ICAM3.5, ICAM3.5mutRUNX, ICAM3.3, ICAM3.3mutRUNX, AMLcons) or polyclonal antisera against CD209 (Control antibody, Cnt Ab) or RUNX1 proteins (R-3034). The position of RUNX1-containing complexes are shown. Unlabeled competitor oligonucleotides were added at a 100-fold molar excess. **D.** EMSA was performed on the ICAM3.4 oligonucleotide using nuclear extracts from THP-1 cells in the absence (−) or presence of unlabeled competitor oligonucleotides (ICAM3.4, ICAM3.4mutCEBP, C/EBPcons) or polyclonal antibody against CD209 (Control antibody, Cnt Ab) or C/EBPα proteins (α-C/EBPα). The position of C/EBPα-containing complexes are shown. Unlabeled competitor oligonucleotides were added at a 100-fold molar excess. In A–D, EMSA's were performed twice with similar result and a representative experiment is shown. **E.** ICAM-3 promoter-based oligonucleotides with mutated nucleotides in lowercase and their relative positions. **F.** *In vivo* occupancy of the ICAM-3 promoter by RUNX1. Chromatin immunoprecipitation on Jurkat cells was performed with an affinity-purified polyclonal antisera specific for RUNX1 or purified rabbit IgG. Immunoprecipitated chromatin was analyzed by PCR using a pair of ICAM-3 promoter-specific primers that amplify a 234-bp fragment flanking the RUNX-binding sites at −80 and −29. ChIP experiment was performed twice with similar results, and a representative experiment is shown.

Most of the tissue-specific activity of the ICAM-3 gene was retained in the region of the promoter −164/+53 (Fig. 1B). To find the transcription factors involved in the restricted-expression of ICAM-3, gel shift assays were performed with oligonucleotides spanning the region −157/−14 (Fig. 2A). Comparison of the pattern of retarded complexes among distinct hematopoietic cells lines indicated that recognition of the region −93/−14 was cell type-specific (Fig. 2A, C, D). The pattern of binding on ICAM3.3 and ICAM3.5 probes was similar in nuclear extracts from THP-1 and Jurkat cell lines whereas a THP-1-specific retarded complex was observed in ICAM3.4 probe. The pattern of retarded complexes, their absence in K-562 cells, and the presence of putative RUNX-binding sites led us to hypothesize that ICAM3.3 and ICAM3.5 DNA elements were recognized by RUNX factors. Therefore, RUNX1, RUNX3 and CBF-β were overexpressed in COS-7 cells, and the resulting extracts were assayed for binding to ICAM3.3 and ICAM3.5 probes. As shown in Figure 2B, RUNX1/CBF-β and RUNX3/CBF-β recognized the RUNX-binding elements within the ICAM-3 promoter. Moreover, the retarded complexes were inhibited in the presence of the AMLcons oligonucleotide, which contains a consensus binding site for members of the RUNX transcription factor family, and by the anti-RUNX1 antibody R3034 (Fig. 2C) and were not competed in the presence of ICAM3.3 and ICAM3.5 oligonucleotides with mutated RUNX elements (Fig. 2C, E). Altogether, these results demonstrate that RUNX1/CBF-β and RUNX3/CBF-β interact with two sequences located at −80 (R1 element) and −29 (R2 element) within the regulatory region of the ICAM-3 gene (Fig. 2B, C). The ICAM3.4 oligonucleotide contains the sequence TTCTGCAA which matches the consensus C/EBP binding sequence (TTGCGCAA) (Fig. 2E). To determine whether this particular sequence was involved in formation of the myeloid retarded complexes (Fig. 2A), ICAM3.4, ICAM3.4mutCEBP and CEBPcons, an oligonucleotide containing the C/EBP consensus sequence, were used as cold competitors in EMSA experiments with THP-1 nuclear extracts (Fig. 2D). Competition with ICAM3.4mutCEBP oligonucleotide left the pattern of complexes unaffected whereas ICAM3.4 and CEBPcons eliminated the retarded complexes. The specific interaction of C/EBP factors with ICAM3.4 was finally evidenced by the capacity of polyclonal antisera against C/EBPα to inhibit the complexes observed in THP-1 cells (Fig. 2D), thus indicating that in myeloid cells C/EBPα recognizes the TTCTGCAA element at −47 of the ICAM-3 gene regulatory region.

To confirm the *in vivo* occupancy of RUNX factors on the ICAM-3 promoter, chromatin immunoprecipitation assays were performed with Jurkat cells, which exhibit a high level of expression of ICAM-3 (Fig. 1A). The proximal ICAM-3 promoter region, containing both RUNX-binding elements, could be amplified from anti-RUNX1 immunoprecipitated chromatin, whereas no amplification was obtained in the presence of control rabbit immunoglobulins (Fig. 2F). Attempts to perform RUNX3 ChIP were unsuccesfull due to the lack of ChIP-grade RUNX3 antibodies. Therefore, RUNX and C/EBP factors recognize the proximal promoter of ICAM-3 *in vitro* and RUNX recognition can be detected *in vivo* by means of chromatin immunoprecipitation.

### Functional relevance of RUNX binding to the ICAM-3 promoter

**Figure 3:**
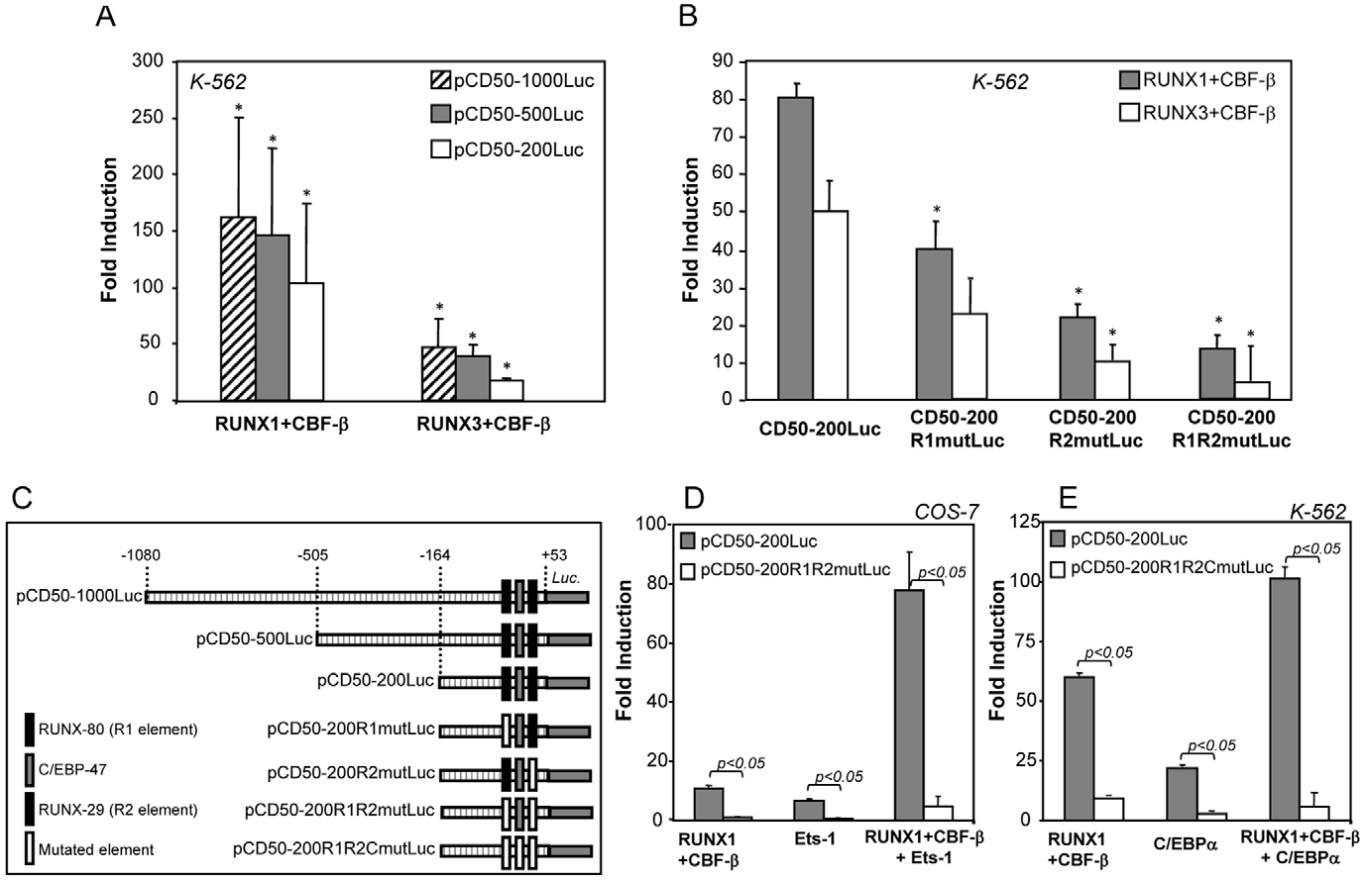
RUNX factors regulate the activity of the ICAM-3 promoter through the recognition of both RUNX-binding sites. **A.** K-562 cells were transfected with 1 µg of the indicated reporter plasmid in the presence of CMV-0 (empty expression vector), pCMV-RUNX1 or pCDNA3-RUNX3, and luciferase activity determined after 24 h. For each individual reporter construct, fold induction represents the luciferase activity yielded by an expression vector relative to the activity produced by a similar amount of CMV-0 plasmid. Data represent mean ± SD of 4 independent experiments using distinct DNA preparations. (*P<0.005 compared with the activity of pCMV-0–transfected cells). **B.** K-562 cells were transfected with 1 µg of the indicated reporter plasmids in the presence of CMV-0, RUNX1/CBF-β or RUNX3/CBF-β expression plasmids, and luciferase activity determined after 24 h. (*P<0.05 compared with the activity of pCD50-200Luc–in the presence of RUNX1/CBF-β or RUNX3/CBF-β, respectively). **C.** Schematic representation of the proximal regulatory region of the ICAM-3 gene and reporter plasmids used for its functional dissection. **D.** COS-7 cells were transfected with the indicated reporter plasmids in the presence of CMV-0, RUNX1/CBF-β or Ets-1 expression plasmids, and luciferase activity determined after 24 h. **E.** K-562 cells were transfected with 1 µg of the indicated reporter plasmids in the presence of CMV-0, RUNX1/CBF-β or C/EBPα42 expression plasmids, and luciferase activity determined after 24 h. In **B, D, E,** for each individual reporter construct, fold induction represents the luciferase activity yielded by an expression vector relative to the activity produced by a similar amount of CMV-0 plasmid. Data represent mean ± SD of 3 independent experiments.

RUNX functional activity is well known to be context- and cell type-dependent and their effect on a given regulatory region varies with the cell lineage and the cellular activation state [22]. Since erythroleukemic K-562 cells are a useful cellular system to illustrate the RUNX-dependent activity of gene regulatory regions (CD36, CD11a) [23], [24], we tested the effect of RUNX protein overexpression on the ICAM-3 promoter activity in this cell line, which is devoid of RUNX1 and RUNX3 [25]. As shown in Figure 3A, overexpression of RUNX1/CBF-β produced a 160 fold increase in the activity of the ICAM-3 promoter construct pCD50-1000Luc. The ICAM-3 promoter transactivation was observed at distinct reporter∶vector ratios (data not shown) and with all the deletions containing the RUNX-binding elements R1 and R2. Transfection of RUNX3/CBF-β also led to a great increase in the activity of the ICAM-3 promoter (47-fold for pCD50-1000Luc) although in all cases the transactivation effect was lower than with RUNX1/CBF-β (Fig. 3A). Then, the effect of mutation of the RUNX-binding sites, either separately or combined, was evaluated within the pCD50-200Luc context. As shown in Figure 3B, mutation of R2 element reduced the transactivation to 30% of the level observed on the wild type promoter, while mutation of R1 element reduced RUNX transactivation twice thus implying that the R2 element plays a more relevant role in RUNX1- and RUNX3-dependent transactivation. Moreover, mutation of both RUNX-binding elements considerably reduced (83% and 85%, p<0.05) the transactivation capacity of RUNX1 and RUNX3 (Fig. 3B, C). Altogether, these results indicate that RUNX factors regulate the activity of the ICAM-3 promoter through interaction with both R1 and R2 RUNX-binding elements.

### C/EBPα and Ets-1 collaborates with RUNX in ICAM-3 transactivation

Sequence analysis and EMSA experiments in the ICAM-3 gene regulatory region suggested that C/EBPα and Ets factors could be implicated in ICAM-3 promoter regulation (Fig. 1C, 2D). Since both factors have been previously reported to collaborate with RUNX [26], [27], we evaluated the influence of Ets-1 and C/EBPα in transactivation experiment (Fig. 3D–E). RUNX1 and Ets-1 transactivated ICAM-3 promoter and mutation of R1 and R2 elements considerably reduced the transactivating capacity of both factors. Co-expression of RUNX1 and Ets-1 produced a considerable increase in the activity of the ICAM-3 promoter (on average 78-fold), and mutation of both R1 and R2 RUNX-binding elements resulted in a complete loss of the collaborative effect. Similar results were obtained in K-562 cells, where the activity of the ICAM-3 promoter in the presence of RUNX1 and C/EBPα was higher than the activity exhibited in the presence of each individual factor, and mutation of RUNX and C/EBP-binding elements abrogated the collaborative effect (Fig. 3E). Altogether, these results demonstrate that the transactivation ability of RUNX1 on the ICAM-3 promoter is enhanced in the presence of either Ets-1 or C/EBPα and indicates that RUNX1 synergizes with Ets-1 in transactivation of the ICAM-3 promoter.

### Modulation of ICAM-3 expression: ICAM-3 is downregulated during monocyte to macrophage differentiation, monocyte transendothelial migration and DC maturation

**Figure 4:**
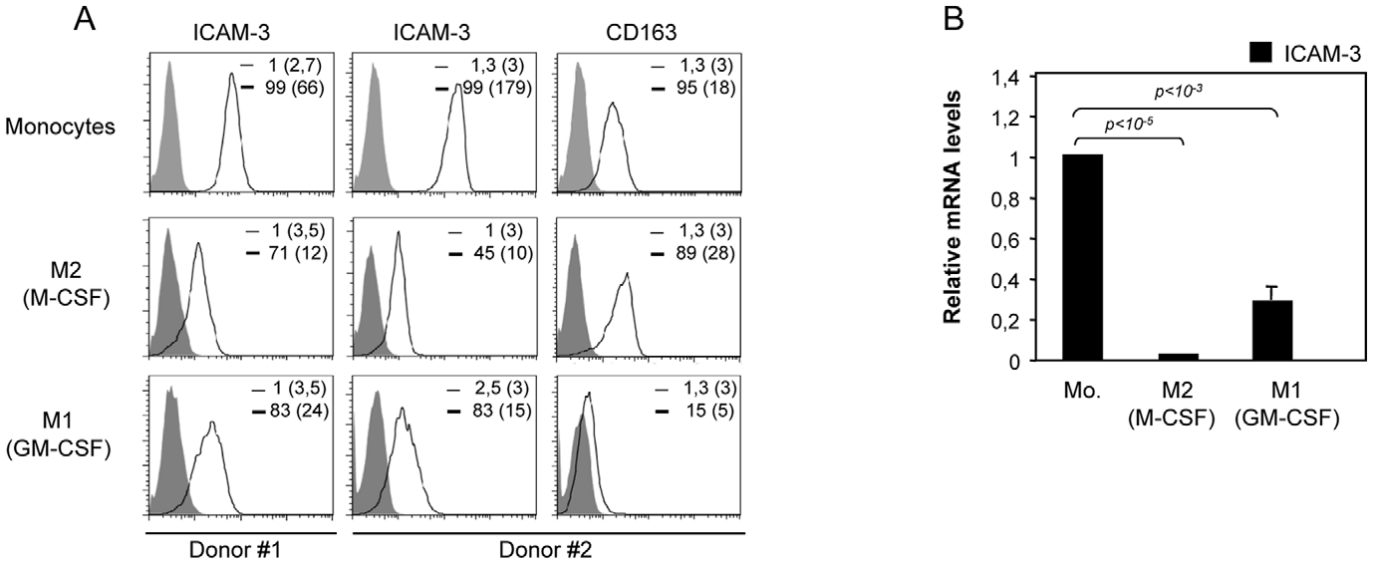
Expression of ICAM-3 during macrophage differentiation. **A.** ICAM-3 expression on monocytes and macrophages differentiated in the presence of either GM-CSF (M1) or M-CSF (M2) during 7 days from two different donors, as determined by flow cytometry (empty histogram). As a control (filled histogram), an FITC-labeled isotype antibody was used. The percentage of marker-positive cells and the mean fluorescence intensity (in parenthesis) are indicated in each case. In donor 2 expression of CD163 is indicated as a control of differentiation. **B.** *ICAM-3* mRNA expression levels on monocytes, and fully differentiated (7 days) M1 (GM-CSF) and M2 (M-CSF) macrophages, as determined by qRT-PCR. Results are expressed as Relative mRNA levels (relative to *GAPDH* mRNA levels and the *ICAM-3* mRNA level on monocytes). Data represent mean ± SD of 3 independent donors.

**Figure 5:**
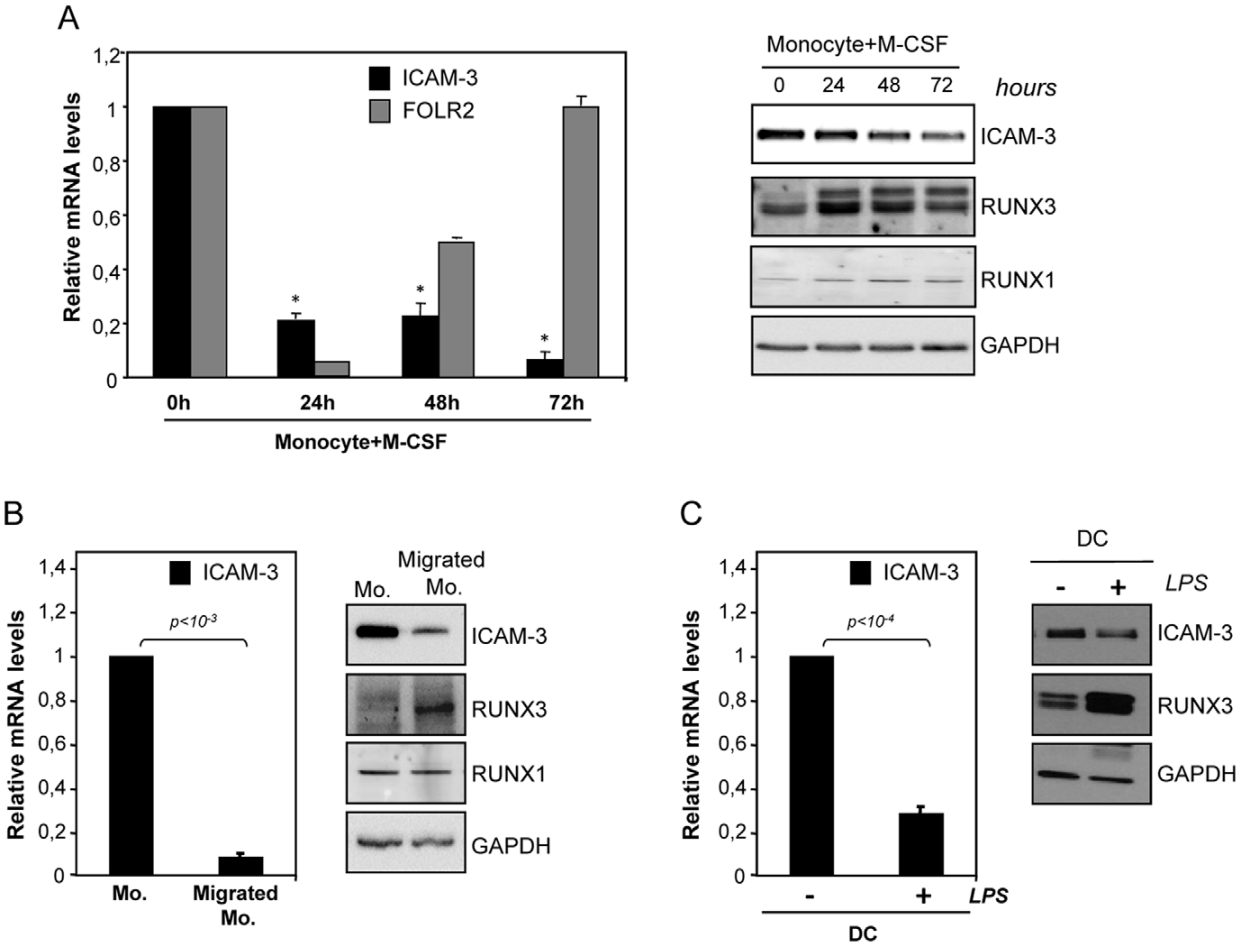
Expression of ICAM-3, RUNX1 and RUNX3 during macrophage differentiation, monocyte transendothelial migration and DC maturation. **A.** Left, *ICAM-3* and *FOLR2* mRNA expression levels along M-CSF monocyte-derived-macrophages, as determined by qRT-PCR at the indicated time points. Results are expressed as Relative mRNA levels (relative to *GAPDH* mRNA levels and the *ICAM-3* and *FOLR2* mRNA level on monocytes). Data represent mean ± SD of 3 independent donors (*P<0.05 compared with ICAM-3 mRNA level of monocytes). Right, ICAM-3, RUNX1 and RUNX3 expression on monocytes and M-CSF-polarized macrophages, as determined by Western blot at the indicated time points. As a control, GAPDH expression levels were also determined. The experiment was performed twice and one of the experiments is shown. **B.** Left, *ICAM-3* mRNA expression levels of monocytes (Mo.) and transendothelial migrated monocytes (Migrated Mo.), as determined by qRT-PCR. Results are expressed as Relative mRNA levels (relative to *GAPDH* mRNA levels and the *ICAM-3* mRNA level on monocytes). Data represent mean ± SD of 3 independent donors. Right, ICAM-3, RUNX1 and RUNX3 expression on monocytes (Mo. and transendothelial migrated monocytes (Migrated Mo.), as determined by Western blot at the indicated time points. As a control, GAPDH expression levels were also determined. The experiment was performed twice and one of the experiments is shown. **C.** Left, *ICAM-3* mRNA expression levels of DC either untreated (−) or treated with 10 ng/ml of LPS during 24 h (+) as determined by qRT-PCR. Results are expressed as Relative mRNA levels (relative to *GAPDH* mRNA levels and the *ICAM-3* mRNA level on untreated DC). Data represent mean ± SD of 3 independent donors. Right, ICAM-3 and RUNX3 expression on DC either untreated (−) or treated with 10 ng/ml of LPS during 24 h (+), as determined by Western blot. As a control, GAPDH expression levels were also determined. The experiment was performed twice and one of the experiments is shown.

The cytokines GM-CSF and M-CSF contribute to macrophage differentiation and polarization [28], and we next analyzed the expression of ICAM-3 in monocytes and fully polarized macrophages differentiated by either M-CSF (M2 macrophages) or GM-CSF (M1 macrophages). In the presence of either cytokine ICAM-3 cell surface expression was greatly diminished, although ICAM-3 levels were lower in M2 (M-CSF) macrophages, which exhibited higher CD163 expression (Fig. 4A) [29]. The scavenger receptor CD163 is a macrophage marker preferentially found on M2 (M-CSF) macrophages [30]. In agreement with the cell surface expression data, *ICAM-3* mRNA levels were also reduced in both M1 (GM-CSF) and M2 (M-CSF) fully polarized macrophages (Fig. 4B), indicating that *ICAM-3* downregulation is linked to monocyte-to-macrophage differentiation. Kinetic analysis revealed that *ICAM-3* mRNA downregulation was initially detected 24 h after M-CSF addition and was maintained thereafter (Fig. 5A), while the M2 (M-CSF) macrophage specific marker *FOLR2* mRNA increased 48 to 72 hours after M-CSF addition [31]. Since M-CSF downregulates monocyte-ICAM-3 expression, RUNX proteins were examined in parallel. While RUNX1 protein expression levels were kept constant, the RUNX3/p44 and RUNX3/p46 isoforms increased 24 h–48 h after M-CSF addition, indicating that the expression of RUNX3 and ICAM-3 are inversely correlated. Evaluation of monocytes during transmigration across an endothelial monolayer was also used to measure the link between RUNX factors and ICAM-3 expression. Migrated monocytes exhibited lower levels of *ICAM-3* mRNA and protein than non-migrated monocytes. By contrast, RUNX3 expression increased after monocytes transmigrate through the endothelium (Fig. 5B). Finally, we extended these findings to the DC maturation process and examined ICAM-3 and RUNX protein levels during LPS-induced DC maturation (Fig. 5C). LPS maturation diminished mRNA and protein expression of ICAM-3 (Fig. 5C) while increased the maturation marker CD83 (data not shown). In agreement with previous reports [32], RUNX3p44 and p46 expression increased during the 24 h LPS-treatment, while RUNX1 levels were undetected (data not shown). These results confirmed that, like in the case of M-CSF-macrophage differentiation and monocyte transendothelial migration, the expression of RUNX3 and ICAM-3 are inversely correlated.

### RUNX3 inhibits ICAM-3 expression in myeloid cells

**Figure 6:**
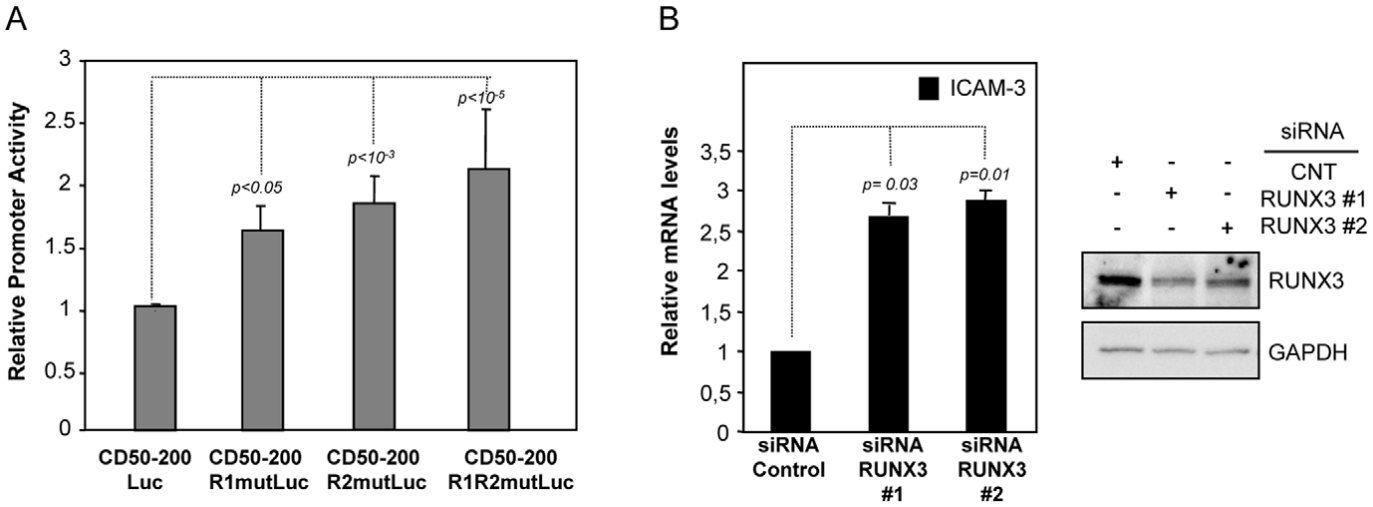
Functional relevance of RUNX-binding sites and influence of RUNX3 on ICAM-3 mRNA expression level in THP-1 cells. **A.** Disruption of the RUNX-binding elements leads to increased ICAM-3 gene promoter activity in THP-1 cells. THP-1 cells were transfected with the indicated reporter plasmids and luciferase activity was determined after 24 h. Promoter activity is expressed relative to the activity produced by the wild-type CD50-200Luc, arbitrarily set to 1, after normalization for transfection efficiency. Data represent mean ± SD of 6 independent experiments using two different DNA preparations. **B.** Knockdown on RUNX3 results in increased ICAM-3 mRNA levels. THP-1 cells were nucleofected with either siRNA for RUNX3 (two different RUNX3 specific-siRNA, siRNA RUNX3#1 and siRNA RUNX3#2) or a control siRNA (siRNA CNT). After 24 hours, total RNA was isolated and *ICAM-3* mRNA was measured via quantitative RT-PCR (left). Results are expressed as Relative mRNA levels (relative to GAPDH mRNA levels and the ICAM-3 mRNA level in control siRNA-nucleofected cells). Data represent mean ± SD of 3 independent experiments. To confirm siRNA efficiency, one-fifth of the cells were lysed and underwent western blotting (right). The western blot was performed twice with similar results and one of the experiments is shown.

The opposed regulation of RUNX3 and ICAM-3 expression that we had observed during macrophage differentiation and DC maturation led us to hypothesize that RUNX3 negatively regulates ICAM-3 expression. To test this hypothesis, we selected the myeloid THP-1 cell line because its transfectability allowed us to analyze the influence of mutations of the RUNX-binding sites on the ICAM-3 promoter activity and the effect of knocking-down RUNX3 on ICAM-3 expression. Disruption of R2 element produced a significant (90%, p<10^−3^) increase in the activity of the ICAM-3 promoter, demonstrating that preventing RUNX binding to the R2 element increases the activity of the ICAM-3 promoter in ICAM-3-expressing myeloid cells. The effect of disrupting the R1 element was lower (60% increase), while the simultaneous mutation of R1 and R2 RUNX-binding elements increased promoter activity twice (p<10^−5^), thus demonstrating that RUNX negatively regulates ICAM-3 promoter activity in myeloid cells (Figure 6A). To definitively prove the direct influence of RUNX3 on ICAM-3 expression, ICAM-3 mRNA expression level was assessed by a knockdown approach on ICAM-3-expressing THP-1 cells. Nucleofection of two distinct RUNX3-specific siRNA in THP-1 cells, which reduced RUNX3 levels by more than 50% (Fig. 6B), led to an increase of the *ICAM-3* mRNA levels as determined by quantitative RT-PCR (Fig. 6B). Therefore, decreasing RUNX3 expression had a direct impact on the *ICAM-3* RNA levels in THP-1 cells, thus confirming the negative involvement of RUNX3 in ICAM-3 gene expression.

## Discussion

The ICAM-3 adhesion receptor mediates leukocyte-leukocyte interactions and its expression is restricted to hematopoietic lineage cells. The basis for the leukocyte restricted and regulated expression of ICAM-3 has remained so far unknown. We now report that the ICAM-3 leukocyte restricted expression reflects the tissue-specific activity of the ICAM-3 gene promoter. Besides, we show that Ets, C/EBP and RUNX factors control the activity of the ICAM3 gene regulation region, where they bind in a tissue-specific manner, thus suggesting that these factors might contribute to its tissue-restricted activity. In fact RUNX1, RUNX3 and C/EBP bind *in vitro* to the ICAM-3 gene promoter, which is occupied *in vivo* by RUNX1 in lymphoid Jurkat T cells. Moreover, we have observed that ICAM-3 expression is dramatically downregulated at the early stages of the *in vitro* monocyte to macrophage differentiation process and upon monocyte transmigration across endothelial monolayers. In both circumstances, ICAM-3 downregulation correlates with an enhanced expression of RUNX3, suggesting a negative regulatory action of RUNX factors on ICAM-3 expression. This suggestion was confirmed by the enhanced ICAM-3 gene promoter activity observed after mutation of two proximal RUNX-binding sites and by the enhanced *ICAM-3* mRNA levels in siRNA-RUNX3-transfected THP-1 macrophages. Like in the case of the CD36 gene [23], RUNX factors negatively regulate ICAM- 3 gene promoter activity in THP-1 myeloid cells, while they potentiate the promoter activity in erythroleukemic K-562 cells, thus adding the ICAM-3 gene promoter to the list of gene regulatory regions where RUNX factors exert a context-dependent function (activation versus repression). As a whole, the present report constitutes the first description of the processes where ICAM-3 expression is dramatically modulated and the identification of the transcription factors that regulate ICAM-3 expression.

Unlike ICAM-1, whose expression is highly induced by pro-inflammatory cytokines and is sensitive to the cytokine environment [18], ICAM-3 expression has not been reported before to be dependent on extracellular stimuli. The lack of a murine orthologue for human ICAM-3 has precluded the definition of its *in vivo* physiological role, and its functions during immune and inflammatory responses have been deduced from *in vitro* experiments. In this regard, and through its interaction with LFA-1, ICAM-3 on lymphoid T cells mediates homotypic aggregation [8], T cell co-stimulation [13] and the initial scanning previous to immunological synapse formation [12]. Within the myeloid lineage, ICAM-3 is the predominant co-stimulatory ligand for LFA-1 on human blood DC [33], since blocking anti-ICAM-3 antibodies are potent inhibitors of DC-stimulated allogenic responses. This has led to the hypothesis that ICAM-3 is the most relevant LFA-1 ligand during the initial stages of the DC-T lymphocyte interactions [12], [33]. Accordingly, the higher levels of ICAM-3 on M1 (GM-CSF) macrophages compared to M2 (M-CSF) macrophages is consistent with the enhanced ability of M1 macrophages to induce antigen-specific and allogenic T cell proliferation [28] (data not shown). Regarding DC, *ICAM-3* mRNA levels are reduced during the LPS-induced maturation of human DC [34], whereas RUNX3 is transiently upregulated [32] (Fig. 5C), further supporting the inverse correlation observed during monocyte to macrophage differentiation and monocyte transendothelial migration.

ICAM-3 is highly expressed on peripheral blood monocytes and very rapidly downregulated upon differentiation into macrophages or following transendothelial migration (Fig. 4 and 5). Similar to the role of ICAM-3 during lymphocyte recruitment [9], the high expression of ICAM-3 on monocytes may contribute to foster the entry of other leukocytes into tissues. During leukocyte transendothelial migration ICAM-3 is highly polarized towards the trailing uropod, where it interacts with LFA-1 of the following cell, acting as a guide for new waves of leukocytes into the tissues [9]. The head to tail recruitment of chain migrating cells has also been described during *Dictyostelium discoideum* chemotaxis and appears to be a general mechanism that cells use to amplify chemotactic responses [35]. Whereas the reason for ICAM-3 downregulation remains speculative, the guiding function of ICAM-3 would end up once leukocytes complete the transendothelial migration, a time at which we have observed ICAM-3 expression to be virtually undetectable.

The loss of ICAM-3 in differentiating or transmigrating monocytes/macrophages has other potentially alternative implications. It has been previously described that the identity of the LFA-1 ligand mediating T lymphocyte co-stimulation determines the resulting cytokine profile, with the Th1 cytokine TNF-α been more highly produced upon LFA-1 recognition of ICAM-2 or ICAM-3 and higher levels of IL-10 produced upon interaction with ICAM-1 [36]. Consequently, monocytes/macrophages expressing different levels of ICAM-3 would exhibit different T cell polarization capabilities. Since RUNX factors control ICAM-3 expression, RUNX could contribute to the T cell polarization ability of monocytes/macrophages. In addition, it is well established the role of RUNX3 in T cell polarization, as it enhances T-bet dependent IFNγ secretion from T lymphocytes while silences IL-4 expression [37]. Therefore, RUNX factors would modulate immune response polarization by regulating gene expression in both T lymphocytes and antigen-presenting cells.

In summary, we have presented evidences of a previously unnoticed regulatable expression of ICAM-3 in human monocytes and shown that RUNX and C/EBP factors are involved in controlling the expression of this integrin ligand. Previous studies have demonstrated that RUNX1/3 factors regulate the expression of integrins like LFA-1, CD11c, CD49d [38], [39], implying that RUNX factors simultaneously control the expression of the integrin LFA-1 and its ligand ICAM-3. Therefore, the ability of RUNX factors to control immune response polarization can be accounted for, at least partially, their ability to regulate the expression of molecules that mediate critical adhesive interactions during immune responses.

## Materials and Methods

### Cell culture and treatments

The human cell lines THP-1 (monocytic leukemia), K-562 (chronic myelogenous leukemia), Jurkat (T cell lymphoma), EA.hy926 (umbilical vein), and the monkey kidney fibroblast-like cell line COS-7 were obtained from the American Type Culture Collection (ATCC, Rockville, MD) [23], [24], [39]. The melanoma cell line BLM was provided by Goos Van Muijen (Radboud University, Nijmegen, The Netherlands) [39]. THP-1, K-562, Jurkat, BLM cells were cultured in RPMI supplemented with 10% foetal calf serum (FCS), at 37°C in a humidified atmosphere with 5% CO_2_. EA.hy926 and COS-7 cells were maintained in DMEM supplemented with 10% FCS. Human monocytes were purified from peripheral blood mononuclear cells as described [31]. GM-CSF (M1), M-CSF (M2) monocyte-derived macrophages and monocyte-derived dendritic cells (DC) were generated as described [23], [30]. Phenotypic analysis was carried out by direct immunofluorescence using FITC-labeled anti-ICAM-3 (BD Biosciences) and FITC-labeled anti-CD163 monoclonal antibodies (MBL International Corp, MA). For transendothelial migration assays, thick collagen gels [40] were coated with EA.hy926 cells grown as a monolayer. 2×10^6^ monocytes suspended in RPMI 10% FCS with 10 ng/ml of M-CSF were plated and allowed to transmigrate for 24 hours. Migrated cells were recovered after 30 min. of collagenase treatment.

### Transfections, plasmids, and site-directed mutagenesis

COS-7, K-562, BLM and Jurkat cells were transfected with Superfect (Qiagen, Hilden, Germany), and THP-1 cells were transfected using DEAE-dextran. In reporter gene experiments, the amount of DNA in each transfection was normalized by using the corresponding insertless expression vector (CMV-0) as carrier. Each transfection experiment was performed at least three times with different DNA preparations. Transfection efficiencies were normalized by cotransfection with the pCMV-βgal plasmid, and β-galactosidase levels determined using the Galacto-Light kit (Tropix, Bedford, Massachusetts).

The ICAM-3-based reporter gene constructs pCD50-1000Luc, pCD50-500Luc and pCD50-200Luc were generated by PCR amplification of the −1080/+53, −505/+53 and −164/+53 fragments of the CD50 promoter with oligonucleotides 5′-CCCAAGCTTCCTTGGAATGCAGTGACC-3′, 5′-CCCAAGCTTAGGTCAAGATGAGAGGAGGC-3′, 5′-CCCAAGCTTGGAGACTCAGCAGTGCTGG-3′, 5′-CCGCTCGAGGACAGAGGAAGGTGCCTTCC-3′, and the resulting fragments were cloned into HindIII/XhoI-digested pXP2 plasmid, which contains the firefly luciferase cDNA [41].

Site-directed mutagenesis was performed on the ICAM-3 promoter construct pCD50-200Luc using the QuikChange System (Stratagene, La Jolla, CA). For mutation of the RUNX-80, RUNX-29 elements, the oligonucleotides RUNX1-80mutS (5′- GCATCCCCTTCCACCTGTAGGCCTGGGCAAGG -3′), RUNX1-80mutAS (5′-CCTTGCCCAGGCCTACAGGTGGAAGGGGATGC-3′), RUNX2-29mutS (5′-GCAATCTTAACCGCTACACTTCCGTCGCAGTGGGAGG-3′), RUNX2-29mutAS (5′-CCTCCCACTGCGACGGAAGTGTAGCGGTTAAGATTGC-3′), were used, and the resulting plasmids were termed pCD50-200R1mutLuc and pCD50-200R2mutLuc. Generation of the pCD50-200R1R2mutLuc plasmid, where the RUNX-binding sequence RUNX-80 and RUNX-29 are mutated, was accomplished by site-directed mutagenesis on the pCD50-200R1mutLuc plasmid using the oligonucleotides RUNX2-29mutS and RUNX2-29mutAS. Generation of the pCD50-200R1R2CmutLuc plasmid, where the C/EBP-binding sequence C/EBP-47 is mutated was accomplished by site-directed mutagenesis on the pCD50-200R1R2mutLuc plasmid using the oligonucleotides CEBP-47mutS (5′- GCAGTCCCCAGACTTCTGGTCTCTTAACCGCTGTGC) and CEBP-47mutAS (5′- GCACAGCGGTTAAGAGACCAGAAGTCTGGGGACTGC-3′). DNA constructs and mutations were confirmed by DNA sequencing.

### 5′-Rapid amplification of cDNA ends (5′-RACE)

The identification of transcriptional start sites of ICAM-3 was performed by 5′-RACE assays using FirstChoice RLM-RACE kit (Ambion) with cDNA from Jurkat and THP-1 cells. The 5′ end of human CD50 was amplified by PCR using the forward 5′RACE outer primer (5′-GCTGATGGCGATGAATGAACACTG- 3′) and the reverse gene specific primer CD50-SP4 (5′-CGACTGTTGCCAGTCACGTT -3′) located at the ICAM-3 exon 2, and the PCR product was subjected to a nested PCR amplification using 5′RACE inner primer (5′-CGCGGATCCGAACACTGCGTTTGCTGGCTTTGATGAAA-3) and the reverse gene specific primer CD50-SP3 (5′-AGCAGAGAGCACAGGGTTCT-3′), located also at the ICAM-3 exon 2. The nested PCR product was cloned into the pCR2.1TOPO (Invitrogen) and sequenced.

### Electrophoretic mobility shift assays (EMSA)

Nuclear extracts were prepared according to Schreiber et al. [42] and EMSA was performed as described [38]. For antibody inhibition experiments, R-3034 (polyclonal antiserum against RUNX1, generously provided by Dr. N. A. Speck), α-C/EBPα (sc-61X from Santa Cruz Biotechnology) or α-CD209 (DSG2, polyclonal antiserum against DC-SIGN) [43] were incubated with the nuclear extracts at 4°C for 30 minutes before the addition of the probe.

### Western blot

Total cell lysates were obtained in RIPA buffer containing 2 µg/ml aprotinin, antipain, leupeptin, and pepstatin. Ten µg of cell lysate was subjected to SDS-PAGE and transferred onto a PVDF membrane (Millipore, Bedford, MA). Protein detection was carried out using antibodies against ICAM-3 (clone sc-8268, Santa Cruz Biotechnologies, Santa Cruz, CA), RUNX1 (39000, Active Motif, Carlsbad CA), RUNX3 (R3-5G4, MBL International Corporation) and β-actin (Sigma-Aldrich, UK) or GAPDH (clone sc-32233, Santa Cruz Biotechnologies, Santa Cruz, CA).

### Quantitative real time RT-PCR

Oligonucleotides for *ICAM-3*, *FOLR2*, and *GAPDH* were designed according to the Roche software for quantitative real time PCR, and RNA was amplified using the Universal Human Probe Roche library (Roche Diagnostics). Assays were made in triplicates and results normalized according to the expression levels of GAPDH. Results were obtained using the ΔΔCT method for quantitation.

### Chromatin immunoprecipitation (ChIP) assays

ChIP was performed using the EZ ChIP assay kit (Upstate Biotechnology, Lake Placid, NY) as described [44]. ICAM-3 promoter was detected by PCR using the oligonucleotides 5′- GGAGACTCAGCAGTGCTGG-3′ and 5′-GTACCATGGTGGCCATTCTG-3′, which together amplify a 234 bp region between positions −164 and +70. Immunoprecipitating antibodies included rabbit polyclonal antisera against human RUNX1 (39000, Active Motif, Carlsbad CA) and purified rabbit IgG as a control (Serotec, Oxford UK).

### Knockdown assays

THP-1 cells were nucleofected with siRNA for RUNX3 or a negative control (s2467, s2469, #1, siRNA Silencer Select, Ambion Applied Biosystems, Austin, TX), using the Cell Line Nucleofector kit V (Amaxa, Cologne, Germany). After nucleofection, cells were kept in culture for 24 h, and one-fifth of the cells were lysed and subjected to Western blot for protein detection. Total RNA was isolated from the rest of nucleofected cells and subjected to real time-PCR for detection of *ICAM-3* and *GAPDH* mRNA.
